# Bibliographical Notices

**Published:** 1847-02

**Authors:** 


### BIBLIOGRAPHICAL NOTICES.

*An Address to the Class of the Medical College of Georgia, at the opening of the Session of* 1846-7. *Containing a sketch of the improvements in Medicine during the present century.* By L. A. Dugas, M. D., Professor of Physiology and Pathological Anatomy. 8vo. pp. 16. Augusta, 1846.

*A Lecture on Practical Education in Medicine and on the course of Instruction at the New York Hospital; delivered at the Hospital, Nov.* 3, 1846. By John Watson, M. D., one of the Surgeons of that Institution. 8vo. pp. 19: New York, 1846.

*Introductory Lecture delivered before the Class of the Medical Department of the Saint Louis University. Session of* 1846–7. By Henry M. Bullitt, M. D. Professor of Physiology and Pathology. 8vo. pp. 19. Saint Louis, 1846.

*An Introductory Lecture on the Reciprocal Obligations of the Medical Profession and Society.* By John P. Harrison, M. D. Professor of Materia Medica in the Medical College of Ohio. Delivered November 2, 1846. 8vo. pp. 28. Cincinnati, 1846.

*Lecture Introductory to the Course of Materia Medica and Pharmacy in the Medical Department of Pennsylvania College. Session of* 1846–47. By Henry S. Patterson, M. D. (With a motto from Weinhart.) 8vo. pp. 20. Philadelphia, 1846.

*An Introductory Lecture delivered before the Class of Jefferson Medical College, November* 5, 1846. By Robert M. Huston, M. D. Professor of Materia Medica and General Therapeutics. 8vo. pp. 24. Philadelphia, 1846.

*Introductory Lecture.* By Charles D. Meigs, M. D. Professor of Midwifery and the Diseases of Women and Children in Jefferson Medical College. 8vo. pp. 24. Philadelphia, 1846.

*Lecture Introductory to a Course on Obstetrics and the Diseases of Women and Children. Delivered October* 30, 1846. By GUNNING S. BEDFORD, M. D. Professor of Obstetrics and the Diseases of Women and Children in the University of New York. 8vo. pp. 26. New York, 1846.

*Introductory Lecture to the Course on the Principles and Practice of Surgery in the Medical Department of Pennsylvania College. Session of* 1846-47. By DAVID GILBERT, M. D. 8vo. pp. 18. Philadelphia, 1846.

*An Introductory Lecture delivered before the Class of the Baltimore College of Dental Surgery, at the Session of* 1846-47. By A. WESTCOTT, A. M., M. D. Professor of Operative and Mechanical Dentistry. 8vo. pp. 32. Baltimore, 1846.

Lectures introductory to courses on the various departments of medicine are often "abstracts and brief chronicles of the time." The professor does not feel himself necessarily restricted to the subject which he has to illustrate during the course. Common custom permits him to digress, and occasionally it happens, that he leaves the domain of medicine and wanders into other departments of science. We have heard of an introductory lecture to a course of Institutes of Medicine on 'Hunting,' which might, however, be regarded hygienically; and we have ourselves listened to a learned but somewhat imaginative discourse on the Natural History of the 'Seventeen year Locust'—*Cicada septendecim*—the prolegomenon to a course of lectures on the Theory and Practice of Medicine.

The lectures before us are somewhat discursive. A few are strictly introductory to the departments taught by their respective authors. Others touch more or less directly on the agitating question of *medical reform*—as it is commonly designated; and as few but reformers have much zeal in the cause, most of the writers are on that side of the question. Moral courage is demanded of any one who feels satisfied with the things 'that be,' and declares so openly; and it is too much the habit with the over-zealous—and therefore, too often intemperate, hasty, and injudicious—to consider those who do not belong to the movement party 'arrogant,' as if the charge did not apply rather to active agitators, who are dissatisfied with arrangements over which they have no direct agency—although such agency would doubtless be most grateful to them.

It has been often said, that the present is eminently an age of improvement; and with this sentiment we fully accord. The profession, within the last few years, has advanced at a more rapid pace than probably at any former period of the same duration in medical history. We believe that there never were in the ranks of the profession so many who have had the benefit of medical instruction in the schools; and we doubt whether there was, at any time, so great a proportion of readers; and whether in all respects the profession was as respectable. Improvements may be, and are, suggested; some of them feasible; but most of them not; and it must ever be borne in mind, that modifications of educational systems which have been wisely suggested, and sustained by long experience, ought not to be made hastily; and that every great change is an experiment, which in a government—or system of twenty-nine governments—like ours, may not be found as satisfactory in practice as it seems to be sound in theory.

Let the youth intended for the medical profession have—as we remarked in our last number—the intellectual and moral training that befits the well educated gentleman;—let boards of examiners see that the candidate for practice has the necessary qualifications, and there can be no well founded cause of complaint on the score of insufficiency of attainments. The standard of qualification must ultimately be determined by such examining boards; and if they do their duty, the wildest reformer can see no necessity for periodical meetings of the profession with the expressed intention of pointing out, and attempting to rectify, defects, some of which are doubtless real; but even if real, their repeated promulgation is calculated to do more harm to the profession in its connexion with the people than all the vagaries of the empiric, and all the follies of homœopathy and mesmerism, against which certain journalists appear to direct their shafts, whilst they pass over in silence the employment of secret agents by members of our own profession, who, by publishing the results of their experience with such agents, become the open abettors of quackery, and scarcely more elevated in the moral scale than the quacks themselves. Reform—it appears to us—is as imperiously demanded on the part of many of those already in the profession, as of such as are preparing to enter within its pales. We know it will be said, that the imperfectly educated, the low in the social scale, are more apt to embrace those very practices, and such may be the fact; still truth must compel us to admit, that examples are occasionally given by those who occupy high places, and whose conduct becomes the more injurious in consequence. Instances of this kind have been recently sufficiently and lamentably notorious, and may suggest interesting reflections to the members of the Convention who are about to congregate in this city.

But:—to the lectures before us:—

That of Dr. Dugas professes to be a sketch of the improvements in medicine during the present century. As in many similar productions, there is in it, we think, an overstrained eulogy of the medical profession. In the latter part of the following quotation, too, the exception appears to us to be brought forward as the rule; for so far as our own observation has extended, the mass of aspirants for medical honors have been impelled to enter the profession solely by their desire to obtain through it an honorable mode of subsistence.

"In Europe, where the Church, the Bar, the Army and the Navy are the high roads to political as well as to social preferment, we find the members of these professions almost exclusively derived from the wealthy and aristocratic classes of society. In our country, those who are ambitious of political station usually select the Bar. The medical profession is, on the contrary, made up of those whom neither wealth, nobility nor political ambition, prompts to seek any other than scientific distinction—that which can neither be bought with gold nor acquired by hereditary transmission, but which must flow from personal merit alone. It cannot be conceded that many enter this profession as a means of earning a livelihood; but it is an innate love of knowledge, a desire to look into the mysteries of nature, which prompts them, unconsciously perhaps, to select this in preference to other pursuits in which less scientific research is necessary." p. 6.

The progress of medical knowledge in all its departments, during the present century, is a field which might be tilled most productively; but the author before us has scarcely broken the surface; so that the announcement on the title-page is not satisfactorily fulfilled. Not much opportunity—it is true—is afforded for detail in a discourse of the kind, but many of the materials might, we think, have been better selected.

We do not know what amount of authority the lecturer has for the following broad assertion.

"Modern medicine has been peculiarly successful in facilitating the treatment of infantile diseases. At a time when the patient's testimony was the only source of information, a correct knowledge of the diseases of those deprived of language or of intelligence, as children and idiots, was unattainable; whereas, with the aid of physical signs and the *method of exclusion*, by which all organs ascertained to be in a healthy state are excluded from farther observation, there are few, very few, cases in which any difficulty will be experienced in the formation of a correct diagnosis." p. 14.

Or for the following.

"There is no disease in which the usefulness of our profession is more signally illustrated than that commonly called bilious fever, for whilst, if left to the unaided efforts of nature it will almost invariably terminate fatally, it now rarely if ever does so under early and skilful medication." p. 15.

Or for the following.

"The Medical College of Georgia may justly claim the merit of having been the first to promulgate the great reformation in the treatment of paroxysmal fevers." p. 16.

The lecture "on practical education in medicine" by Dr. Watson naturally dwells, but in no measured terms of eulogy, on the New York Hospital, which, if we were to judge from the Announcements and other advertisements of the Medical Department of the University of New York, affords to the student of medicine facilities not enjoyed elsewhere, and of which we would infer from the same sources he is not slow to avail himself. Under this impression, indeed, students have been induced—we had almost said *enticed*—to visit New York, when they have been surprised to find scarcely any in attendance. So late, indeed, as the winter of 1845-6—according to the New

York Journal of Medicine—not more than twenty of the students actually took the Hospital ticket ; and in the lecture before us Dr. Watson grievously deplores their scanty attendance.

"There were probably," he says, "between six and seven hundred medical students in this city (New York) during the last winter. I was on duty in the second surgical department of this hospital during the greater part of that time, and strange to say, *it was a rare thing to see the face of a single student in any of my wards during the whole of my attendance.*"

With what propriety, then, can the array of cases of sickness treated in that institution be adduced by any Medical College as an incentive to the medical student for preferring New York to Philadelphia, when in the latter city there are not only opportunities for clinical instruction, but these opportunities are actually embraced by numerous students. During the last official year—we speak from authority—not fewer than *one hundred and forty-six* students took the ticket at the Pennsylvania Hospital; and from Jan. 1846 to Jan. 1847—the University of Pennsylvania requiring their candidates for graduation to have attended one course of hospital instruction—not fewer than *two hundred and eighty-three.*

Dr. Watson properly urges the importance of clinical instruction, and who denies it? But it is an interesting question, whether it be to the welfare of the patient or to the advantage of the student that *masses* shall congregate daily in the wards, disturb the febrile and inflammatory, and examine individually, and in succession, the physical signs in thoracic and other diseases. There can be, from the philanthropist but one answer we apprehend to the question ; and hence the clinical teacher is compelled to select cases—as was long the custom at the Philadelphia Hospital and is still at the College clinics—for illustration in the amphitheatre,—the student having, at the same time, ample opportunity for witnessing the surgical and general management of the hospital.

So far, then, as facilities are afforded and embraced, there would seem to be no comparison between the advantages actually derived from clinical attendance by the students of Philadelphia and of New York.

The author of the lecture gives a brief history of clinical instruction in this country and elsewhere, into which we cannot follow him. We have only space for one or two comments and rectifications. He is not quite as cosmopolitan as we could desire. Not only the hospital to which he is attached, but other institutions in the city of his residence, occupy, we think, too high a place in his thoughts. For example, he says:

"The cause of education in our well-appointed colleges—and *especially in the rival schools of this city*, (rivals be it ever hoped only in their ability for usefulness,) is every year becoming more and more elevated."

*Quod est demonstrandum.* We pause at least for the evidence.

Dr. Watson is a reformer—doubtless the advocate of a judicious reform—as we were wont to hear of the advocates of a judicious tariff. The following sentiment appertains to many as well as to himself.

"Gentlemen, the medical institution which first comes up to the reasonable demands of the profession in this respect, and, possessing the facilities, requires of its graduates practical acquirements equal to those at present enjoined upon the students of European schools, will find its interest in the measure." p. 8.

We do not believe in this. Such an institution may be able to exclaim with Francis the first, "*tout est perdu sauf notre honneur*," or it may have to submit to its benches being filled by a "select few." We have now in mind a distinguished literary institution, in which it was determined to "raise the standard," and to render its highest honours difficult of attainment. This was done; and whilst the graduates of other institutions were annually numerous, this distinguished school did not confer its highest degree at any time on more than half a dozen; and in some years, we believe, not a single candidate presented himself. It may be said, that they who succeeded would be more respected and successful than the alumni of other institutions where graduation was more easy; but we have had no evidence of this; on the contrary we do not know a single case in which marked advantage has accrued to the graduate for the toil which he had to undergo for this more elevated collegiate distinction. Let a department of higher mathematics be established in our colleges, and even if filled by a La Place or a Gauss, it might be attended by a few pupils; whilst the benches appropriated to lower mathematics under an ordinary teacher would be filled to overflowing. A certain, or rather uncertain, amount of knowledge is admitted by all to be requisite; but the general feeling — erroneous we grant it to be — is that high literary and scientific distinction is not necessary, and may be injurious in the transaction of the business concerns of life; hence we not unfrequently see the learned and accomplished physician dismissed, and the lowest empiric consulted in his place. It is strange, but not less true, that an individual ordinarily—and even more than ordinarily—intelligent will consent to confide in the professional judgment of one whose opinions on any other matter he would contemn.

On the subject of "College Clinics," Dr. Watson dwells at some length, properly urging, that "to dispensary service, when confined to the management of such affections as legitimately belong to it, there is no direct objection;" but college dispensaries should never be held up as sufficient to supply the place of hospitals.

The college *cliniques*, he remarks, were first started in a neighbouring city, "where hospital privileges had been so much restricted as to be of little service to the winter students." In this he is altogether misinformed,—if he applies the remark to Philadelphia.

"As an expedient for supplying this deficiency, they were unquestionably acceptable on the same principle that half a loaf is better than no bread. They were at first called by their true name; but this attracting ltitle notice was soon altered. The example thus set, and the title thus assumed, were copied by the schools of other places."

Dr. Watson does not here do justice to the city of his choice. We believe, that much of the credit that attaches to the college clinics belongs to it, and that the very name was applied to the "clinical" service at the University of New York before it was adopted here.

"The *clinique epidemic* of the colleges, as my friend Dr. Bell has happily expressed it," [we do not see wherein the happiness lies,] "was last winter at its height. The students, one and all, were affected by it, and the hospitals were forsaken."

Certainly not in Philadelphia. The observation may and doubtless does apply to New York, but not to this city.

The lecturer adds—

"It is to be presumed, ere this, the disease has materially abated. Such I am told, is the fact in Philadelphia, where it first broke out. The University of Pennsylvania, and some of the other schools there have already abandoned their dispensaries, and are now supplying their second course students with hospital tickets at their own expense."

Some one has evidently been gulling the worthy author. There is not a word of real history in this quotation. There has been no abandonment of their clinics by other schools, and consequent supplying of tickets to the hospitals gratuitously. The clinic of the Jefferson Medical College was never in so flourishing a state, and so extensively useful. One other clinic has been added to the list--that of the Franklin College. The Pennsylvania College has never had a regular clinic, and gives—as it did last year—to second class students tickets to the Pennsylvania Hospital; but no such thing has been done by the University of Pennsylvania, which—if it has abandoned the *name* of college clinics, professes to teach—*mutato nomine*—" *demonstrative medicine*" on one day of the week, and "*demonstrative surgery*" on another! It would be difficult to find any where a sentence in which so much error—doubtless unintentional—is contained in so small a compass.

Our opinion of hospital attendance for *masses* has already been expressed, and as *masses* must be supplied in the best mode that is practicable, we do not hesitate to affirm, as our conviction, that the introduction of " College Clinics"—we use the term generally appropriated to them, and see no valid objection to it—is the most important single addition to medical educational resources that has been proposed in recent periods.

" Gentlemen," says Dr. Watson, " if I know my own mind, I have no disposition to draw invidious comparisons, or to speak of the relative advantages of rival cities or of rival institutions." p. 12.

Why then does he exclaim, after having made the erroneous statements referred to above :—"and if hospital attendance there [meaning in Philadelphia,] is beyond measure superior to their misnamed cliniques, [he had before shown that both were desirable, and had not instituted an " invidious comparison,"] what are we to think of the facilities for practical instruction enjoyed by students within the walls of the New York Hospital?"

We do not see the *sequitur.* If the hospital attendance in Philadelphia be better than the college clinics, what are the facilities for practical instruction enjoyed by students within the walls of the New York Hospital? Such appears to be the proposition, which we are not Cocker enough to solve.

We have no doubt of the value of the New York Hospital, and of the entire honesty of purpose of the author of the lecture before us; yet it must be admitted by the best friends of the Institution, that the picture of it given by him *savours* of hyperbole.

"You may," he observes, "in other countries find larger hospitals, but none presenting a greater variety of acute and important diseases; you may find in other hospitals abler teachers, but none so [?] willing as we have been to give you our time and services for nothing. You may find in some few other institutions greater opportunities for autopsic [a vile word] examinations; you may find in the cabinets of foreign societies, more valuable pathological collections; you may, in other cities, even find larger libraries than ours; but look for all these together in any other hospital either at home or abroad, and you will look for them in vain [?] *I say it without fear of contradiction, you will not find a single hospital to compare with this,—not one that contains within itself so many advantages for both theoretical and practical study as the New York Hospital.*" p. 19. Shame, then, on the profession of New York!—Shame, on the Professors of the two "rival schools," the course of education in which "is becoming every year more and more elevated!" Shame on the six or seven hundred medical students in the city during the last winter—that in the second surgical department, during the greater part of the winter, "it was a rare thing to see the face of a single student."

The peroration of the lecturer is judicious and wholly unobjectionable. It is well worthy of being retained in the recollection of the clinical student.

"During the coming winter, it will be well for every medical student here, but more particularly for such as are not prepared to remain in town after the close of the college session, to visit the hospital daily. Let nothing prevent you from attending at the hour set apart for the purpose.

Come not merely with your ears open, as students often do.

Come not to gaze about the wards with idle curiosity. But rather, come like philosophers, with all your faculties awake, for examining, comparing, judging, and thinking for yourselves; and working, too, for yourselves, at every opportunity.

Pick out your own cases, and study these, and note these for yourselves. Do not attempt to listen to all that is said with the view of remembering it:—but listen well to what is said of the cases on your own list, and see if it be true. Attempt not too much.

Do not fall into the peurile fondness of hunting after strange sights, rare diseases, and great cases. The dressing of an ulcer, the setting of a bone, the treatment of a pleurisy, pneumonia, or fever,—these, and such as these, should be your study here. Striking cases you may look at; but the business that should fix your attention, is that which best prepares you for the daily business of the profession.

Finally, avoid that morbid appetite for surgical operations, so long magnified and so much over-rated. Surgery is a good thing, a useful, an excellent thing in its way; but too much of it is a great evil. And the sooner you find out this for yourselves, the better for your patients. p. 19.

Dr. Bullitt's lecture is his maiden effort at the Saint Louis University. It contains much that is interesting and generally well expressed. "My primary allegiance," he remarks, "is to the general cause of medical science, and then to the cause of the medical department of the Saint Louis University;" and he expresses to his class a hope, that he may be able so to discharge the duties of teacher in the institution, as to make his "labours tributary to the common good of our common calling." Such we have little doubt will be the case.

We had marked one or two passages for extract and comment, but we cannot find space for them. This same circumstance will prevent us from giving more than a passing notice of the other introductories before us.

The lecture of Dr. Harrison inquires into the obligations of the medical profession to society, and the obligations of the public to the medical profession; and is an interesting discourse.

That of Dr. Patterson indicates a cultivated intellect. The style is vigorous, tinctured somewhat with the German, and perhaps with Carlylism, which we abhor; but it is never mystical, and always sententious.

The lectures of Dr. Huston and Dr. Meigs were delivered before the class of Jefferson Medical College—the largest, we may add, that has ever attended medical lectures in this country; and therefore sufficient to inspire the learned professors with unwonted energy. Dr. Huston's discourse is a scorching and able disclosure of Hahnemannism, clearly expressed—and it ought to convince all, who are susceptible of conviction, that the whole infinitesimal system is founded on folly, and too often practised in imposture.

The discourse of Dr. Meigs is full of the fire and enthusiasm which have ever characterized him. "I acknowledge" he says, "that I am an enthusiastic admirer of my profession. My speech shows it, and my whole past life is a perpetual proof of it. But I love that profession as a ministry, not as a trade."

The following observations on what has been sarcastically termed "book learning," or "book knowledge," from one who is so eminent in the practical exercise of his calling, are a satisfactory answer to those ignoramuses who pretend to despise books.

"Gentlemen, no man can study medicine by himself. He must have help and that help comes from books. I never could have learned why I ought to do thus and so, to keep a lady patient from bleeding to death, or from perishing with convulsions, out of my own primary independent observation and reflection. If I have done so, I thank the fathers, as I thank every good man—whether Greek or Roman, Arabian or European—who in ages past has put upon record the things that have been observed in medicine, and if I know those things I owe that knowledge to them; I could never have learned it of myself. Therefore, I love books. I think there is no good physician without their aid—nor can be none. Instead of disparaging books and authors, I would rather glorify and honour them when meritorious." p. 8.

The lectures of Professors Bedford and Gilbert are strictly introductory to their courses. We would remind the former, who says, that "Doctor Robert Lee, of London, has recently made some valuable contributions on the subject of the nerves of the uterus,"—that the nerves depicted by Dr. Lee have, by a still more recent observer—Mr. Beck—whose memoir has received a medal from the Royal Society of London—been affirmed to be no nerves at all.

From a remark by Dr. Gilbert, Dr. Watson will find, that the Professor of Surgery in the Pennsylvania Medical College has no objection to surgical cases being brought before a class, which is all that is done in the regular "school clinics."

"Without pretending for a moment," he says, "to present, in addition to the regular course, an *adequate surgical clinic*, whenever the character of the case, however, and other circumstances are such as to render it compatible with the feelings and general advantage of the patient, I will present to the class as much practical surgery as can, with propriety, be brought before them. The experience of the two last sessions warrants me in saying to you, that cases of this kind may be expected; which, with the ample surgical and medical clinics of that time-honored institution, the *Pennsylvania Hospital*, will afford you clinical facilities *equal and we believe superior to those of any school in the country*." [!!]

Lastly. It is not many years since the excellent institution—the Baltimore College of Dental Surgery—which has done, and is still doing, so much to elevate the professional character of the dentist—was chartered by the State of Maryland; and already many interesting and useful productions have emanated from it. The lecture of Dr. Westcott may be esteemed one of these. It canvasses whether "dental colleges possess peculiar advantages over any other means of securing a dental education"—determining the question—as might be expected—in the affirmative, and we are not disposed to cavil at the decision.

We are pleased, to observe, that extensive, general, and professional attainments are urged as indispensable to make up the character of an accomplished dentist. Whilst manual dexterity is important, it is wisely maintained, that the operator should be a well-educated gentleman, and that a knowledge of every branch of medical science should enter into the list of his evidences of fitness to do credit to himself, to the avocation which he has embraced, and to the community.

*A Practical Treatise on Inflammation, Ulceration, and Induration of the Neck of the Uterus: with remarks on the value of Leucorrhœa and Prolapsus Uteri as symptoms of Uterine Disease.* By JAMES HENRY BENNET, M. D., etc. etc, 12mo. pp. 146. Lea & Blanchard. Philadelphia, 1847.

The basis of the present work was submitted by the author, a few years since, to the Faculty of Medicine of Paris as a thesis, on his graduating in that University. Subsequently, more elaborated, the essay was published, in parts, in the London Lancet; and afterwards in a yet more extended form, as a separate publication, of which the present is a reprint.

From the brief autobiography given in the Preface, we learn that the author spent seven years in the Saint Louis, La Pitié, and Salpetrière Hospitals of Paris, first as pupil, and then as "interne" or resident physician.

"Under such circumstances," says the author, "I cannot, certainly, be reproached with not having matured my opinions. In the first instance, they were formed after I had long enjoyed very great opportunities of seeing uterine disease. They have since been considered over and over again, and have stood the test of several years additional experience."

"Some of the views which I bring forward will, I believe, be found original,—at least, if I can trust the results of my bibliographical researches. I have also many details of great interest and importance to present, with reference to the various modes of *treatment* in inflammation, ulceration, and induration of the uterine neck adopted by the Paris physicians and surgeons—details which will, I believe, be new to most of my readers. Having carefully watched, during a great length of time, the effects of the treatment followed by the eminent Parisian practitioners, with whom the knowledge of this form of disease recently originated, and that under the most favourable circumstances—as their pupil or assistant—I have been able, I hope, to form a correct estimate of the comparative value of the different agents they employ. I have thus, I am also inclined to think, learnt how to avoid the exclusiveness which most of them show in the choice of their therapeutic agents."

One can scarcely repress a smile at the confidence with which many of the young members of our profession express their opinions, after a residence of a few years, or as it sometimes happens, of a few months, in Paris, and of which the preceding extract is no very bad example. The natural enthusiasm of youth, operated upon by the example and habits common, to a great extent, in the French metropolis, are perhaps a sufficient apology for this self-complacency, until time chastens the one, and better example corrects the other.

We have looked through Dr. Bennet's *brochure* with some interest to find the "original" views which he deems so important without discovering them, although we have found much in it to approve, and we are inclined to think that his "bibliographical researches" have not been remarkably extensive, even among Parisian authors, to say nothing of British and German.

The subjects embraced in this treatise are of great importance, and we regret to say, generally too little understood by those entrusted with the treatment of such complaints; this, rather than any novelties contained in the book, or that we have to offer, claims from us more than a passing notice.

The author asserts that inflammation of the neck of the uterus, with its sequelæ, ulceration and induration, "is an exceedingly common affection"—and "is the principal cause, also, of several morbid states which are generally, if not always, studied independently of any such origin; as, for instance, prolapsus of the uterus and leucorrhœa."

That inflammation of the neck "is the principal cause" of prolapse of the uterus, is not very clear to our mind, but that it is often mistaken and treated for the latter affection, we know from abundant experience—no mistake indeed is more common.

"With reference to leucorrhœa," says the author, "indeed, I have ascertained, to my complete satisfaction—firstly, that, setting aside cancerous disease, in the very great majority of adult females who have been exposed to sexual intercourse, a confirmed leucorrhœal discharge, whatever may be its nature, is accompanied by inflammation of the neck of the uterus; secondly, that this inflammation seldom exists long without producing ulceration; and, thirdly, that ulceration is *always* accompanied by more or less engorgement (swelling, with or without induration) of the substance of the uterine neck."

We have no doubt of the correctness of these propositions; they are true as far as they go; but they do not go far enough, as it regards leucorrhœa. That inflammation of the vagina, and often of the neck of the uterus, precedes leucorrhœa, no one will deny; and we are prepared to admit with Dr. Bennet that, after no long time, ulcerations of the cervix uteri frequently occur; but this does not account for *uterine leucorrhœa,*—in which the discharge comes from the *cavity* of the uterus.

The author makes what he considers "a fundamental and most important distinction between inflammations and ulcerations which occur in the uterine neck of females who *have never conceived*, and those which take place in the same region in females who *have conceived*—that is, who have either miscarried or borne children." In the consideration of these diseases, he carries this division throughout the book; and in doing so, we cannot doubt that he magnifies unduly the influence of conception on the permanent condition of the parts. Those who bear children, and especially those who suffer frequent miscarriages, are undoubtedly most prone to inflammation and its consequences in the sexual organs, if we except prostitutes, with whom such complaints are almost universal, whether they have conceived or not. It is rare to meet with a woman has been long "on the town" who has not more or less disease of the neck of the uterus, although she may never have suffered from any of the forms of syphilis; nor is it rare to find individuals who have conceived, and even borne one or more children, in whom the condition of the neck of the uterus has returned in its physical condition very nearly to the virgin state—to a condition in no respect less healthy than before conception.

The following fact noticed by the author is one of great importance in practice, and often not appreciated by the practitioner.

"The size and length of the cervix uteri vary considerably in different females—a fact which must necessarily be taken into consideration if we wish to appreciate the existence or non-existence of engorgement, or morbid increased volume, of the organ. Indeed, these physiological (anatomical) variations are so great, that were we to allow ourselves to be guided by size alone, as appreciated by the toucher or speculum, we should, undoubtedly be often misled, and induced to suppose that disease existed when it did not. In reality, a very voluminous healthy cervix uteri is perfectly compatible with entire freedom, even from uneasy sensation. The difference in length of that part of the cervix uteri which projects into the vaginal cavity, is evidently owing principally, to the vagina being implanted, as it were, at different heights on the cervix, so that in some females it is merely a few lines in length, whereas in others it is an inch and a half, or more. This physiological elongation of the cervix uteri may, it appears, be carried to such an extent, that its free extremity reaches the orifice of the vulva. Dr. Henning, in his essays on uterine diseases, lately published in 'The Lancet,' mentions several curious cases of the kind."

The following observations on the subject of hypertrophy of the cervix accords so fully with our own experience, that we cannot refrain from calling the attention of the profession to their importance.

"If the hypertrophy is considerable and general, the prolapsus of the cervix is constant, the irritation great; and ulceration and abundant leucorrhœa are nearly always present. If inconsiderable, or limited to one region of the cervix, the surface of the organ may be free from the disease, and the uterus may prolapse only after long standing, or walking or great fatigue of any kind. These are by no means uncommon states. Indeed, I have no hesitation in saying, that a very large proportion of the cases, both of slight and of severe uterine prolapsus, which are met with in practice, and for which pessaries are so improperly used, are the result of chronic hypertrophy of the uterine cervix."

We would even go further than the author, and say that a large number of the cases treated as prolapsus are either simple inflammation of the cervix or engorgement of the uterus, which result in ulceration or hypertrophy from neglect, and too often from the irritation produced by the improper use of pessaries and other means for the cure of simple prolapse proceeding from relaxation of the uterine supports.

The author witnessed the treatment of a great many cases of cancer of the uterus in the Parisian hospitals, and the following is the result of his observations.

"I have hitherto always found cancers of the cervix uteri, whether ulcerated or not, incurable; like cancer in other parts of the economy. In other words, I have never seen an evidently cancerous tumour or ulceration of the cervix, respecting the existence and identity of which there was not a shadow of doubt, cured—that is, dissolved, removed, either by local applications or by general treatment."

Of "localized cancer of the cervix," treated by amputation, he witnessed no better results. "The cases in which it (amputation) has been performed successfully, are generally considered to have been merely cases of inflammatory induration of the cervix," in which he thinks deep cauterization is a much safer remedy.

---

*The Dog.* By WILLIAM YOUATT, Edited, with additions, by E. J. LEWIS, M. D. Member of the Academy of Natural Sciences of Philadelphia; of the Philadelphia Medical Society; of the Parisian Medical Society, etc. 8 vo. pp. 403. Lea & Blanchard, Philadelphia, 1847.

To the lovers of the dog, (and who does not love that brave and devoted animal?) this is a most acceptable volume. Not only to the sportsman and the naturalist, but to the physician, and the housekeeper of every grade, the history of this faithful and sagacious animal,—his diseases and his habits,—is full of interest. "The dog, next to the human being, ranks highest in the scale of intelligence, and was evidently designed to be the companion and the friend of man." "Man," says Burns, "is the God of the dog; he knows no other; and see how he worships him. With what reverence he crouches at his feet—with what reverence he looks up to him—with what delight he fawns upon him, and with what cheerful alacrity he obeys him!" An animal that guards us while we sleep, enjoys our pastimes, and watches with almost parental care the tottering footsteps of our children, has strong claims on our benevolence, and it is the duty of those who exact his services to minister to his wants not less in sickness than in health. To do this effectually, they must study his constitution, his habits, his diseases and their remedies. To all such we commend Mr. Youatt's book. The medical reader will be surprised to learn from its pages "how many ills that animal shares in common with the human race;" whilst the general reader will find its pages rich with anecdotes and varied information. No inconsiderable part of this has been supplied by the editor, who has, indeed, performed his part with much care and ability.

The work is beautifully got up by the Philadelphia publishers—paper, typography, binding—every thing is in keeping. The engravings representing the varieties of the dog are by Gilbert & Gihon, and are well executed.

---

*A System of Human Anatomy, General and Special.* By ERASMUS WILSON, M. D. Lecturer on Anatomy, London. Third American, from the third London edition. Edited by PAUL B. GODDARD, A. M., M. D. Professor of Anatomy in the Franklin College of Philadelphia. With two hundred and thirty-three illustrations by Gilbert. 8vo. pp. 610. Lea & Blanchard: Philadelphia, 1847.

Since the publication of the former American edition, a third edition has been issued by the author in London, containing some additions and improvements. The present edition, we are assured by the editor, "is a careful and exact reprint of the English work, with the addition of such other illustrations as were deemed necessary to a more complete elucidation of the text." Thus it will be seen that the value of the present edition is materially enhanced over that of its predecessors, and in its present improved form offers to the student all the assistance that can reasonably be expected from such a work.

---

*Second Annual Announcement of the Medical Institute of Cincinnati, Session of* 1847.

The lecturers of this Association are Drs. Judkins, Woodward, Warder, Kendrick, Vattier, Mendenhall, Raymond, and Stuart; several of whom are advantageously known as contributors to the Medical Journals, and all, we have no doubt, well qualified for the duties they have assumed. The lectures are to commence on the first Monday in March, and continue four months, embracing all the subjects usually taught in summer schools.

We are glad to see our younger brethren in "the Queen City of the West," exerting themselves in increasing the facilities for gaining instruction in their beautiful town. We have ever regarded such enterprises favourably, and can see no reason why the attempt should not succeed in a city of so large a population as Cincinnati, when made in the right spirit and sustained with becoming energy. From the impossibility of carrying on dissections, and of giving demonstrations in anatomy on the recent subject, during warm weather, a full and complete course on all the branches of medical education, such as is necessary to qualify a young man for graduation, cannot be expected, even if it were possible, which no one will pretend, for the student to undergo the fatigue, in hot weather, of following the requisite number of lectures each day to complete the whole in four or even six months but attendance on one or two lectures a day will hardly be too great a tax upon his health, while it will materially assist his comprehension of what he reads; and when the subjects taught are collateral, and not mere repetitions of the winter lectures, great advantage must accrue to the industrious learner.

